# The Association Between Hearing and Physical Functioning in the Atherosclerosis Risk in Communities Study

**DOI:** 10.1093/geroni/igaa057.1711

**Published:** 2020-12-16

**Authors:** Pablo Martinez-Amezcua, Pei-Lun Kuo, Kevin Sullivan, Priya Palta, A Richey Sharrett, Jennifer Schrack, Frank Lin, Jennifer Deal

**Affiliations:** 1 Johns Hopkins Bloomberg School of Public Health, Baltimore, Maryland, United States; 2 National Institute on Aging, Bethesda, Maryland, United States; 3 University of Mississippi Medical Center, Jackson, Mississippi, United States; 4 Columbia University Irving Medical Center, New York, New York, United States; 5 Johns Hopkins University, Baltimore, Maryland, United States; 6 Johns Hopkins University School of Medicine, Baltimore, Maryland, United States

## Abstract

Hearing loss is highly prevalent among older adults and has deleterious effects on health. However, its association with physical functioning is not well defined. We investigated the cross-sectional association between hearing and physical function in 3,339 community-dwelling participants (mean age: 79 years, 59% women) of the Atherosclerosis Risk in Communities Study (ARIC). Hearing was measured by pure-tone average ([dB]) of 4 frequencies [0.5,1,2,4 kHz] and physical function was measured using the short physical performance battery (SPPB), which consisted of 3 performance-based tests (balance, gait speed, and chair stands) each scored ranging from 0-4, resulting in a total possible score of 0-12 (higher scores indicating better physical function). We estimated the association between hearing and physical function using continuous scores for each component of the battery, and the overall SPPB score categorized into high [10-12], intermediate [7-9], and low [≤6]) using ordinal logistic regression models. The SPPB scores were reversed for an easier interpretation of the odds ratios (OR). The category with better physical functions was the reference group for each model. After adjustment for demographics and comorbidities, poorer hearing (+10 dB in PTA) was associated with worse physical functioning: OR for lower balance score=1.17, 95% CI [1.08, 1.26]; OR for lower gait speed score=1.15, 95%CI [1.06, 1.25]; OR for lower chair stand score=1.07, 95% CI [1.04, 1.11]; and OR for lower overall SPPB category=1.15, 95%CI [1.07, 1.24]. Hearing loss is associated with poorer physical functioning, highlighting the potentially negative impact of hearing loss on mobility at older ages.

